# Coexistence of Right Tubal Ectopic Pregnancy and Parasitic Fibroid in Anterior Abdominal Wall and Broad Ligament: A Rare Surgical Encounter

**DOI:** 10.1155/crog/7028476

**Published:** 2025-11-03

**Authors:** Saba Mubbashir, Olanike Bika

**Affiliations:** Department of Obstetrics and Gynaecology, The Rotherham NHS Foundation Trust, Rotherham, UK

**Keywords:** broad ligament fibroid, ectopic pregnancy, fertility, laparoscopy, leiomyoma, myomectomy, parasitic fibroid, pelvic adhesions

## Abstract

**Background:**

Parasitic fibroids are rare extrauterine leiomyomas that can arise spontaneously or following prior uterine surgery, particularly with morcellation. Their coexistence with ectopic pregnancy is exceptionally rare and presents a unique surgical challenge. We report a rare case of concurrent right tubal ectopic pregnancy, broad ligament fibroid and anterior abdominal wall parasitic fibroid.

**Case Presentation:**

A 30-year-old woman (P0 + 2) presented with acute right iliac fossa pain and a serum *β*-hCG level > 9000 IU/L. She had a history of two prior laparoscopic myomectomies. Transvaginal ultrasound suggested a right adnexal ectopic pregnancy with a large posterior uterine fibroid and free pelvic fluid. Laparoscopy revealed an unruptured right tubal ectopic pregnancy, a fibroid in the broad ligament and a separate parasitic fibroid attached to the anterior abdominal wall. A laparoscopic right salpingectomy, excision of the parasitic fibroid and adhesiolysis were performed. Histology confirmed ectopic pregnancy and parasitic leiomyoma. The patient had an uneventful recovery.

**Discussion:**

The simultaneous occurrence of ectopic pregnancy and parasitic fibroids is highly unusual. Prior myomectomies, especially those involving morcellation, may predispose patients to parasitic fibroid formation through iatrogenic tissue implantation. In this case, distorted pelvic anatomy due to adhesions and fibroids may have contributed to tubal implantation of the embryo. This case highlights the rarity of parasitic fibroids and emphasises the importance of preventive measures during myomectomies, such as contained morcellation to avoid implantation of parasitic fibroids. While intraoperative mindfulness is important, parasitic fibroids are an uncommon finding and are not a routine consideration during common gynaecological presentations, such as ectopic pregnancy.

**Conclusion:**

This case illustrates a rare but significant intersection of fibroid pathology and ectopic pregnancy. Awareness of parasitic fibroids in patients with prior fibroid surgery is essential for surgical planning and optimising reproductive outcomes. Further investigation into the pathophysiological mechanisms linking fibroid surgery to altered fertility and ectopic gestation is warranted.

## 1. Introduction

Uterine fibroids are the most common benign gynaecological tumours in women of reproductive age. Most women with fibroids are asymptomatic, and nearly a third of patients have significant symptoms such as dysmenorrhea, menorrhagia, abnormal uterine bleeding, secondary anaemia, pelvic pain and infertility [1, 2].

FIGO classifies fibroids based on their location within the uterus. Submucosal fibroids (Types 0–2) extend into the uterine cavity, with Type 0 being completely pedunculated and Types 1 and 2 having increasing degrees of intramural extension. Intramural fibroids (Types 3–4) are confined to the uterine wall, with Type 3 in contact with the endometrium but not extending into the cavity, while Type 4 remains entirely within the myometrium. Subserosal fibroids (Types 5–7**)** project toward the serosal surface, with Type 5 having significant intramural involvement and Type 7 being completely pedunculated. Other fibroids (Type 8) include cervical, broad ligament and parasitic fibroids. Hybrid fibroids (2–5 classification) span both the endometrial cavity and serosa, requiring a combined classification based on their extent [3].

Traditionally, parasitic leiomyomas are described as unusual variants of pedunculated leiomyomas that, for reasons unknown, exist outside the uterus in the abdominal cavity, surviving by obtaining a blood supply from neighbouring structures. These leiomyomas are linked with nonspecific clinical signs and symptoms and are typically discovered incidentally during surgery for another primary reason [4]. We report a case of rare coexistence of ectopic pregnancy, broad ligament fibroid and parasitic fibroid.

## 2. Case Report

A 30-year-old woman (P0 + 2, previous TOPs) presented with right iliac fossa (RIF) pain for 1 day. She had a history of laparoscopic myomectomy twice. She was not on any contraception. Initial assessment revealed a *β*-hCG > 9000 IU/L. On examination, she was tender in the lower abdomen, and some guarding was noted. She did not have any medical issues, no h/o STIs, was a nonsmoker and was up to date with her smears.

An ultrasound was carried out, which showed no intrauterine pregnancy. Adjacent to the right ovary, there was a well-circumscribed doughnut-shaped mass, consistent with an ectopic pregnancy. A 56 × 47 mm fibroid is seen in the right/posterior aspect of the uterus. Free fluid is seen within the POD and left adnexa. She was noted to be tender on the transvaginal scan.

The patient was counselled regarding surgical management, as medical treatment was unsuitable. She consented to laparoscopic management of ectopic pregnancy.

**Figure 1:**
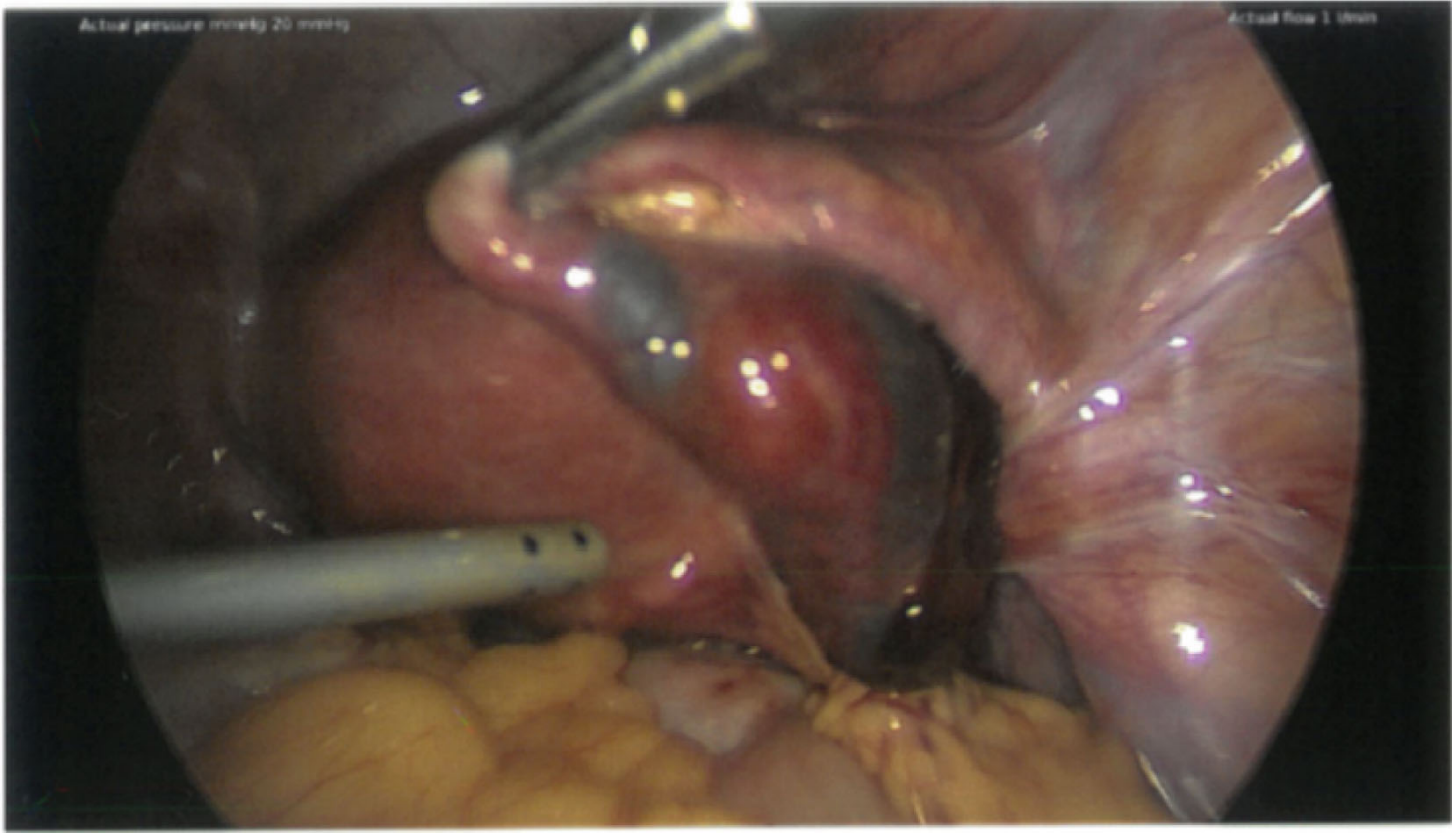
Right tubal ectopic pregnancy.

Intraoperative findings included a right tubal ectopic pregnancy around 5 cm, which was unruptured (Figure 1), with a fimbrial leak (~100 mL hemoperitoneum). Additionally, dense bowel adhesions were found to the posterior uterus, appendix and right adnexa, forming a loop.

**Figure 2:**
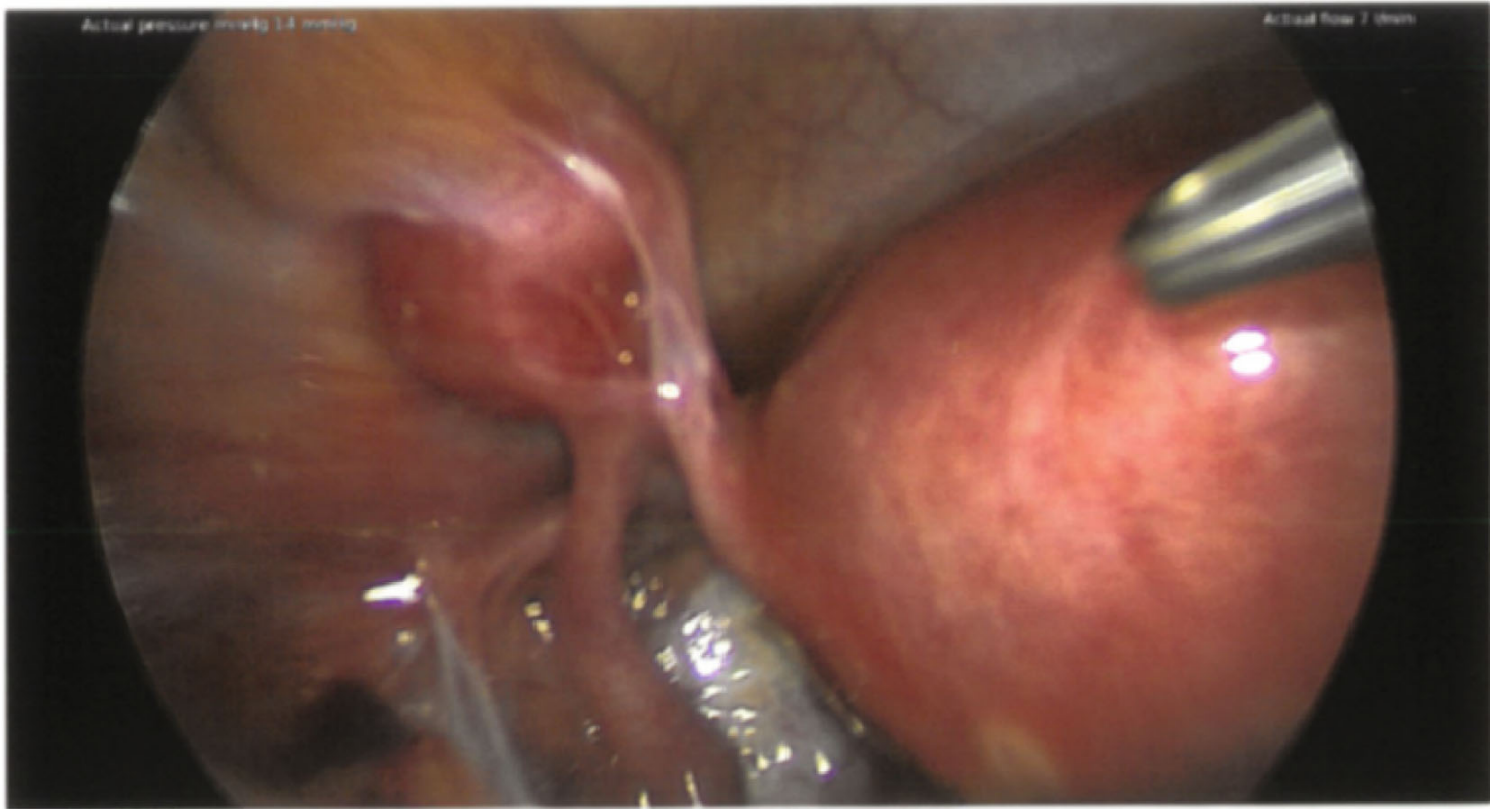
Broad ligament parasitic fibroid on left side.

Multiple uterine fibroids, including a large posterior uterine fibroid (56 × 47 mm) and another fibroid within the broad ligament (Figure 2), are stretching the round ligament and left tube.

**Figure 3:**
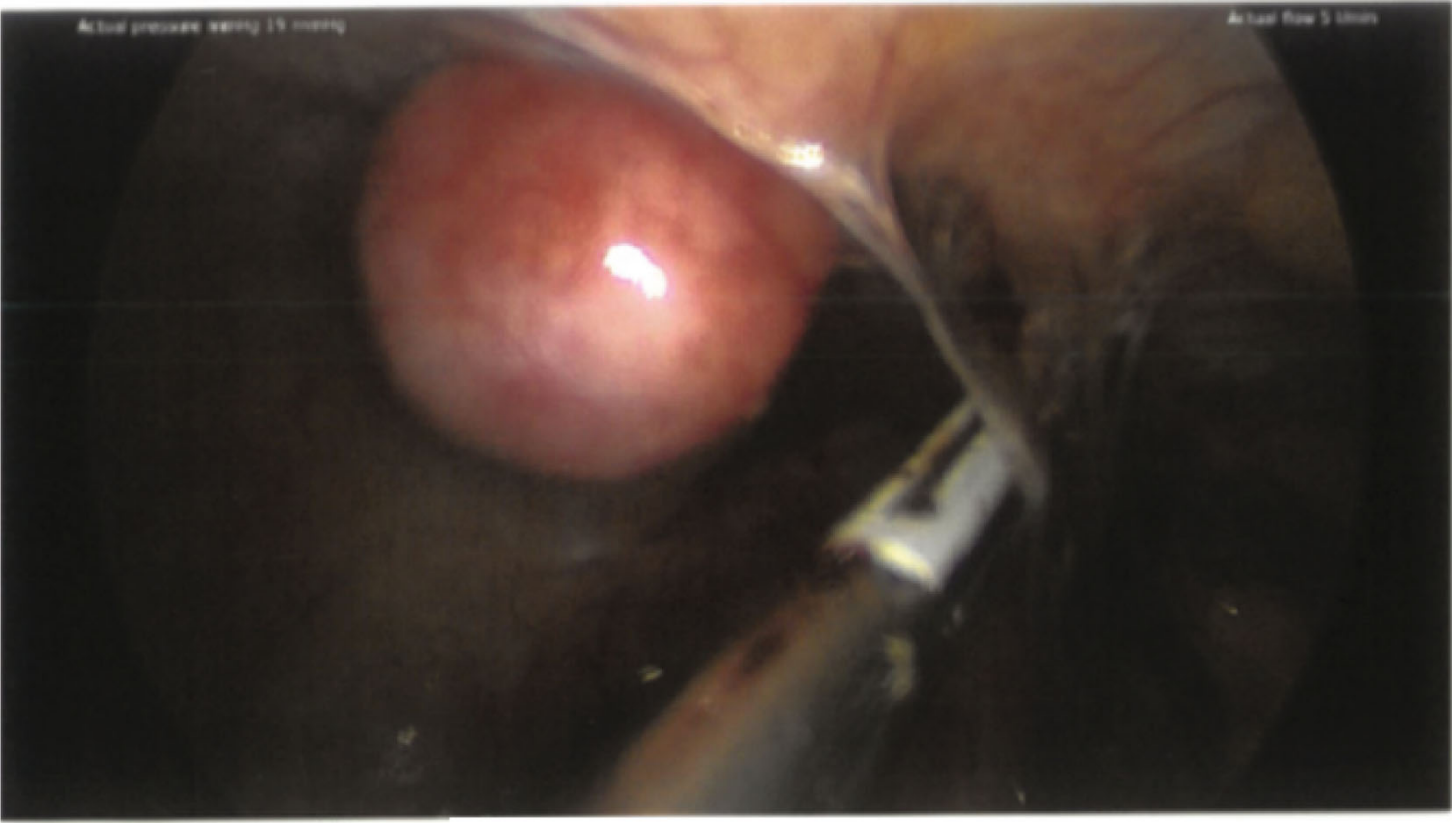
Parasitic fibroid on anterior abdominal wall.

A separate small parasitic fibroid on the right anterior abdominal wall was noted (Figure 3). Both ovaries and the left tube are grossly normal. A laparoscopic right salpingectomy was performed. The parasitic fibroid was excised from the anterior abdominal wall and sent for histology. This small parasitic fibroid was removed as it was accessible to confirm the diagnosis of parasitic fibroid by obtaining tissue for histology. The large intramural fibroid was left in situ in view of not obtaining consent for extensive myomectomy, as the potential risks of such extensive surgery were not discussed preoperatively. We performed extensive adhesiolysis to free the bowel. The broad ligament fibroid was left in situ as it was asymptomatic, and removal would have required extensive dissection with potential risk to the adjacent structures, including the fallopian tube. In addition, preoperative discussion about the potential risks associated with such a procedure was not done; hence, no further intervention was done. We plan to address these in a follow-up appointment.

She had an uneventful recovery and was discharged home on Day 1 post-op. The histology confirmed a right tubal ectopic pregnancy and parasitic leiomyoma.

## 3. Discussion

Ectopic pregnancy and parasitic fibroid coexisting are extremely rare. The fibroid's aetiology is unclear—possibly iatrogenic implantation after prior myomectomy or spontaneous extrauterine development. Parasitic fibroids should be considered in patients with a history of myomectomy, especially if morcellation was used. Imaging may not always distinguish them from peritoneal deposits or endometriotic nodules. Managing preexisting fibroids in the presence of an ectopic pregnancy requires careful intraoperative decision-making to minimise complications and preserve future fertility. There are case reports of the co-existence of broad ligament fibroid along with ectopic pregnancy [5] and tubal pregnancy with tubal leiomyoma [6], but we report the presence of broad ligament fibroid and anterior abdominal wall parasitic fibroid in the presence of an ectopic pregnancy.

Parasitic fibroids are known for atypical clinical presentation, and the clinical features depend on the adjoining visceral structures to which they are attached. They may be asymptomatic and only discovered as an incidental finding at surgery or during radiologic evaluation [7] for other indications, especially if they are small parasitic fibroid nodules. Alternatively, they may be symptomatic, presenting with clinical features such as abdominal swelling or distension, abdominal pain, pelvic pain, dyspareunia, vaginal mass [8] or features of bowel obstruction.

This case highlights how prior myomectomies and the presence of multiple fibroids may have contributed to anatomical distortion, increasing the risk of ectopic pregnancy. Dense bowel adhesions from previous surgeries likely altered tubal mobility and peritoneal dynamics, potentially impairing normal embryo transport. Additionally, parasitic fibroids can develop due to iatrogenic seeding of leiomyoma tissue after laparoscopic morcellation, which may contribute to peritoneal inflammation and subsequent adhesion formation. These structural changes, combined with the absence of intrauterine fibroids affecting endometrial implantation, may have predisposed the patient to implantation within the fallopian tube rather than the uterine cavity, leading to ectopic pregnancy.

Although the woman presented acutely with ectopic pregnancy, the unexpected finding of a parasitic fibroid highlights how a previous myomectomy can alter pelvic anatomy and increase the risk of ectopic pregnancy. In our case, we believe that the adhesions and altered anatomy due to the presence of fibroids likely contributed to an ectopic pregnancy.

## 4. Conclusion

This case highlights the complex interplay between prior fibroid surgery, pelvic adhesions, parasitic fibroids and ectopic pregnancy risk. The rare coexistence of a right tubal ectopic pregnancy, a broad ligament fibroid and a parasitic fibroid adds to the growing evidence that fibroids—especially those outside the uterus—can significantly impact reproductive outcomes. Further research into parasitic fibroid pathophysiology and their long-term effects on fertility is warranted.

## Data Availability

The data supporting the findings of this case report are available from the corresponding author upon request.
